# A differentially expressed set of microRNAs in cerebro-spinal fluid (CSF) can diagnose CNS malignancies

**DOI:** 10.18632/oncotarget.4096

**Published:** 2015-05-28

**Authors:** Alessandra Drusco, Arianna Bottoni, Alessandro Laganà, Mario Acunzo, Matteo Fassan, Luciano Cascione, Anna Antenucci, Prasanthi Kumchala, Caterina Vicentini, Marina P. Gardiman, Hansjuerg Alder, Mariantonia A. Carosi, Mario Ammirati, Stefano Gherardi, Marilena Luscrì, Carmine Carapella, Nicola Zanesi, Carlo M. Croce

**Affiliations:** ^1^ MVIMG, The Ohio State University, Columbus, OH, USA; ^2^ Dept. of Genetics and Genomic Sciences, Icahn School of Medicine at Mount Sinai, New York, NY, USA; ^3^ Dept. of Medicine (DIMED), Surgical Pathology & Cytopathology Unit, University of Padua, Padua, Italy; ^4^ UOSD of Clinical pathology, Regina Elena Institute, Rome, Italy; ^5^ Lymphoma & Genomics Research Program, IOR Institute of Oncology Research, Bellinzona, Switzerland; ^6^ IOSI Oncology Institute of Southern Switzerland, Bellinzona, Switzerland; ^7^ ARC-NET Research Centre, University and Hospital Trust of Verona, Verona, Italy; ^8^ Dept. of Pathology, Regina Elena Institute, Rome, Italy; ^9^ Dept. of Neurological Surgery, The Ohio State University, OH, USA; ^10^ Dept. of Anesthesiology, Sandro Pertini Hospital, Rome, Italy; ^11^ Dept. of Neurological Surgery, Regina Elena Institute, Rome, Italy

**Keywords:** microRNA, cerebro-spinal fluid (CSF), brain tumors, biomarkers

## Abstract

Central Nervous System malignancies often require stereotactic biopsy or biopsy for differential diagnosis, and for tumor staging and grading. Furthermore, stereotactic biopsy can be non-diagnostic or underestimate grading. Hence, there is a compelling need of new diagnostic biomarkers to avoid such invasive procedures. Several biological markers have been proposed, but they can only identify specific prognostic subtype of Central Nervous System tumors, and none of them has found a standardized clinical application.

The aim of the study was to identify a Cerebro-Spinal Fluid microRNA signature that could differentiate among Central Nervous System malignancies.

CSF total RNA of 34 neoplastic and of 14 non-diseased patients was processed by NanoString. Comparison among groups (Normal, Benign, Glioblastoma, Medulloblastoma, Metastasis and Lymphoma) lead to the identification of a microRNA profile that was further confirmed by RT-PCR and in situ hybridization.

Hsa-miR-451, -711, 935, -223 and -125b were significantly differentially expressed among the above mentioned groups, allowing us to draw an hypothetical diagnostic chart for Central Nervous System malignancies.

This is the first study to employ the NanoString technique for Cerebro-Spinal Fluid microRNA profiling. In this article, we demonstrated that Cerebro-Spinal Fluid microRNA profiling mirrors Central Nervous System physiologic or pathologic conditions. Although more cases need to be tested, we identified a diagnostic Cerebro-Spinal Fluid microRNA signature with good perspectives for future diagnostic clinical applications.

## INTRODUCTION

Numerous efforts have been addressed to identify diagnostic and prognostic biomarkers for Central Nervous System (CNS) neoplasms but none of them has found a standardized routine clinical application [1]. Few tissue biomarkers can predict prognosis in only subsets of specific tumor histotypes: IDH1 in diffuse gliomas, 1p19q co-deletion in anaplastic oligodendrogliomas, MGMT methylation in glioblastomas, MYC family members amplification in medulloblastoma, K1AA1549-BRAF fusion gene in pilocytic astrocytomas, EGFR mutation in medulloblastomas and metastasis, etc. [2–6].

Metastasis to the brain often arise from lung, breast, skin, kidney and gastrointestinal tract primary tumors. In 16% of cases, they represent the first evidence of malignancy requiring further clinical, radiographic, and/or histologic studies that cannot always achieve a definitive diagnosis [7–8].

Thus, diagnosis still relays on patients’ clinical features and imaging techniques (MRI and CT). However, non-neoplastic lesions of the CNS may be radiologically and clinically mistaken as tumors: benign lesions are not always distinguished from malignant tumors, and imaging characteristics often underestimate the degree of malignancies [9]. Hence, the definitive diagnosis of brain lesions requires histologic examination of multiple samples obtained by either brain biopsy, or brain stereotactic biopsy or open surgery [10]. Biopsies, beside the peri-operatory complications, show some limitations: sampling error can lead to false negative diagnosis or to misdiagnose non-homogeneous lesions [10, 11].

Therefore, it is essential to identify new biomarkers to integrate the prognostic predictivity of old tests and improve medical diagnostic and prognostic resources through non-invasive means.

MicroRNAs (miRNAs) are short non-protein coding RNAs that function as key regulators of diverse biological processes through regulation of gene expression [12]. Emerging evidence indicates that microRNAs play an important role in the development of human cancers, where they affect the level of expression or the activity of tumor suppressor, oncogenes and other signaling molecules.

Expression profiling has shown that microRNAs signatures differentiate normal from tumor tissues and also correlate to histopathology and prognosis [13, 14]. Importantly, microRNAs can also discriminate the tissue of origin of metastatic lesions [15–19].

Recently, microRNAs have been found in almost all kind of biological fluids, including Cerebro-Spinal Fluid (CSF). Because of their high stability and easy detection by RT-PCR, microRNAs could be the ideal diagnostic and prognostic biomarkers [20, 21].

The aim of our study was to find new biomarkers that could aid in the diagnosis of CNS malignancies in order to avoid bioptic surgical intervention. To reach our goal we analyzed 82 CFS samples by NanoString, and validated a microRNA profile that can differentiate between and among some classes of CNS tumors.

## RESULTS

CSF samples from 34 patients with CNS benign and malignant tumors (Gliomas, Ependymomas, Meningiomas, Glioblastomas, Medulloblastomas, Breast and Lung cancer Metastasis to the brain, primary Lymphomas) and from 14 patients without any malignant, or benign lesion, or degenerative disease, affecting the CNS, were collected at the Regina Elena Institute of Rome, Italy, and at The Ohio State University, OH, USA (Tab.1).

Total RNA was extracted and all 82 samples were processed at NanoString (NanoString Technologies) as described in the material and methods paragraph.

Samples were divided into 7 groups (Normal, Benign, Glioblastoma, Medulloblastoma, Breast Metastasis, Lung Metastasis and primary CNS Lymphoma) for data analysis, and comparisons among groups were performed. As shown in Table 1, some patients had CSF withdrawals at different time points. In these cases the mean expression of the different time points for each miR was calculated and considered for comparisons.

**Table 1:** Samples’ description

Diagnosis	Number of Patients	Number of samples per Patient
Normal	14	1 per patient
Glioma	9	1 per patient
Ependimoma	2	1 per patient
Meningioma	4	1 per patient
Glioblastoma	4	1 in 1 patient 3 in 1 patient 6 in 1 patients 7 in 1 patient
Medulloblastoma	3	1 in 1 patient 6 in 1 patient 7 in 1 patient
Lung cancer Metastasis	4	1 in 3 patients 3 in 1 patient
Breast cancer Metastasis	5	1 in 3 patients 4 in 1 patient 6 in 1 patients
Lymphoma	3	1 per patient

Table 1 shows the number of cases (Number of Patients) for each diagnosis (Diagnosis), associated with the number of sample per case collected (Number of samples per Patient). CSF samples from 34 patients with CNS benign and malignant tumors (Gliomas, Ependimomas, Meningiomas, Glioblastomas, Medulloblastomas, Breast and Lung cancer Metastasis to the brain, primary Lymphomas) and from 14 patients without any malignant, or benign lesion, or degenerative disease, affecting the CNS.

We compared Normals to all groups, the Normal group against every single group separately, the Glioblastoma group versus the Medulloblastoma, and the Lung Metastasis group versus the Breast Metastasis group. We also compared all the malignant (Glioblastoma, Medulloblastoma, Metastasis) groups against the Benign and Lymphoma group.

We selected microRNAs that were showing a significant differential expression among groups in most comparisons (miR-451, -223, -125b, -711, -935, -92a) and miR-664, and -205 to differentiate between lung and breast CNS metastatic lesions.

Only mir-451, -223, -125b, -711 and -935 were validated by single RT-PCR. Fold change differences among groups and significance were calculated. Table 2 shows fold changes and p-Values of each validated microRNA for those comparisons that were significantly differentiating among groups. MicroRNAs showing a Ct value close or equal to 40 were considered not expressed. Not significant comparisons are not reported in Table 2.

**Table 2:** Significant Comparisons among pathology groups

	Up-regulated Group	Group2	Fold Change (2^−(ΔΔCt))	*p*-Value
**miR-451**	Benign	Normal	503.49	< 0.001
	Glioblastoma	Normal	187.76	< 0.001
	Medulloblastoma	Normal	62.86	0.002
	Metastasis	Normal	447.45	< 0.001
				
**miR-711**	Benign	Lymphoma	2.45	0.058
	Glioblastoma	Lymphoma	8.77	< 0.001
	Medulloblastoma	Lymphoma	5.48	< 0.001
	Metastasis	Lymphoma	3.40	< 0.001
	Glioblastoma	Medulloblastoma	1.60	0.018
**miR-935**	Any other group	Glioblastoma(not expressed)		
	Any other group	Medulloblastoma(not expressed)		
	Any other group	Lymphoma(not expressed)		
	Metastasis	Benign	1.80	0.007
	Lung Metastasis	Breast Metastasis	1.69	0.006
**miR-223**	Glioblastoma	Medulloblastoma	3.19	0.031
	Glioblastoma	Normal	98.05	< 0.001
	Medulloblastoma	Normal	37.24	< 0.001
	Benign	Normal	22.20	0.003
	Metastasis	Normal	32.34	< 0.001
	Metastasis	Lymphoma	5.60	0.040
	Glioblastoma	Metastasis	3.03	0.018
**miR-125b**	Medulloblastoma	Glioblastoma	3.91	0.001
	Medulloblastoma	Normal	222.93	< 0.001
	Glioblastoma	Normal	57.00	< 0.001
	Medulloblastoma	Lymphoma	14.61	< 0.001
	Metastasis	Normal	48.45	< 0.001
	Metastasis	Benign	9.02	< 0.001
	Medulloblastoma	Metastasis	4.6	0.001

This Table describes the significant comparison that differentiated among pathology groups (Normal, Benign, Glioblastoma, Medulloblastoma, Metastasis: Lung and Breast) for each validated microRNA. Comparisons are reported together with the corresponding fold changes and p-Values with the exception of those comparison in which the entire group had RT-PCR Ct value close or equal to 40 (not expressed).

To further confirm our findings and, to verify that the identified CSF microRNAs were originating from cancer cells, we performed *in situ* hybridization with each validated miR's LNA probe on FFPE sections of Meningioma, Glioblastoma, Medulloblastoma, Breast and Lung Metastatic lesions and normal adjacent tissue. In situ hybridization experiments, although not sensitive in terms of fold change differences, reflected RT-PCR results.

**Figure 1:**
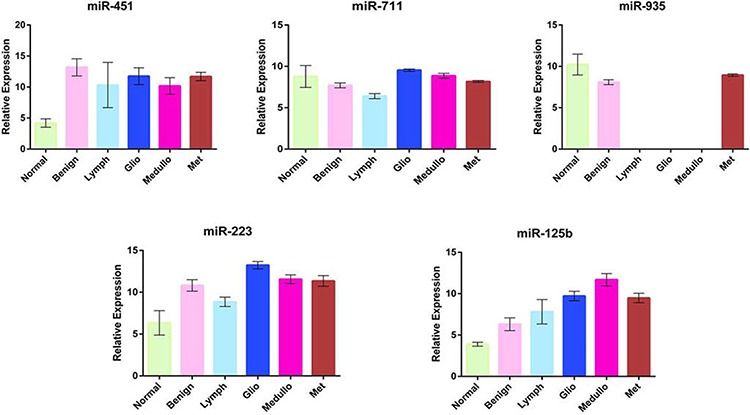
RT-PCR plotted results RT-PCR results are plotted on histograms and show the differential expression of each validated microRNA within groups of patients. Means and SDs are reported in the supplementary material (Table S1). Error bars represent the standard error of the mean.

**Figure 2:**
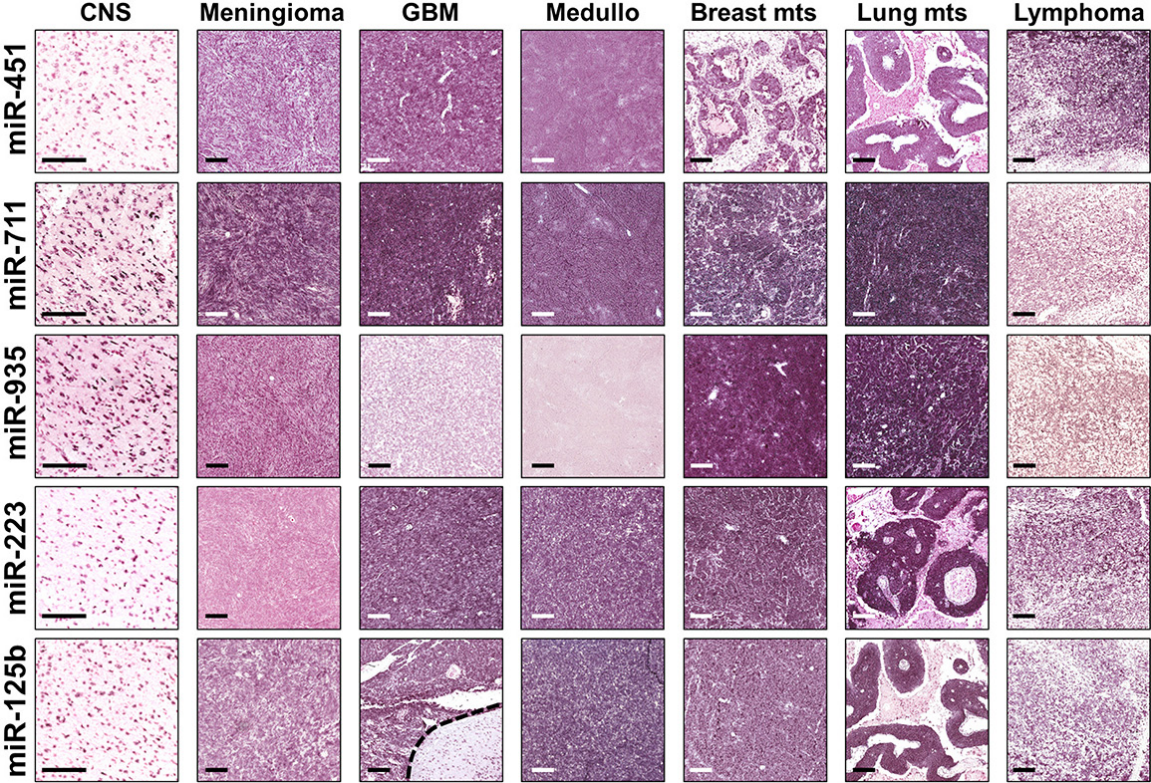
Representative ISH evaluation of miR-125b, miR-223, miR-451, miR-711, miR-935 in tissue sections of primary and metastatic CNS tumors *In situ* hybridization in tissue sections of primary and metastatic CNS tumors demonstrate a significant miRNA expression dysregulation among the different tumor hystotypes. Normal grey matter specimens showed a negative/faint expression for miR-125b, miR-223, and miR-451; on the other hand, normal neurons showed a moderate/strong miR-711 and miR-935 expression. *Columns* denote the different tumor subtypes; *rows* the different miRNAs analyzed. The presence of miRNA is shown by a grainy blue cytoplasmic stain; slides counterstained in fast red. (Scale bars: 200 μm; Original magnifications 10x and 5x).

MiR-451 was significantly down-regulated in Normal and up-regulated in benign and malignant CNS CSF/Tissues. RT-PCR showed a non-significant down-regulation of miR-451 in Normals respect to Lymphomas (Fig. 1). This difference was instead evident with the *In Situ* Hybridization staining (Fig. 2). As shown in Table 2, miR-451 was from 62 to 503 folds less expressed in Normal than the other groups. Thus, a low expression of miR-451 could be used to differentiate patients’ normal CSF from cancer groups.

Similarly, miR-711 was down-regulated in Lymphoma respect to the cancer groups (Fig. 1). The difference was significant when the Lymphoma group was compared to the Glioblastoma, Medulloblastoma and Metastasis groups. The Lymphoma versus Benign comparison showed a borderline significance (*p*-Value = 0.058), but also a 2.45 fold difference in expression. This borderline result can be due to the low number of Lymphoma cases (3 cases) with respect to the Benign cases (9 cases) (Table 2). MiR-711 could be then considered a CNS Lymphoma differentiating microRNA.

Of note is that mir-711 was up-regulated 1.8 folds in Glioblastoma compared to Medulloblastoma (*p*-Value = 0.018).

MiR-935 variable expression has given the most interesting results (Fig. 1).

As clearly shown in Figure 2, which further validated CSF NanoString and RT-PCR data, Glioblastoma and Medulloblastoma tissues did not express miR-935. Lymphoma patients CSF did not expressed miR-935, while tissues were showing a slight stain. This finding obviously mirror the difference between biological fluids and tissue samples.

The absence of miR-935 in CSF can differentiate a Medulloblastoma and/or Glioblastoma and/or Lymphoma from any other neoplastic lesion or from normal tissue/CSF.

MiR-935 was 1.8 fold significantly up-regulated in Metastasis when compared to Benign, but there was no significant difference if compared to Normal. There was no deregulation between the Normal and Benign group.

On the other hand, miR-935 showed a significant (*p*-Value = 0, 006) overexpression in lung compared to breast metastasis.

Two additional microRNAs were part of the confirmed signature: miR125b and miR-223 (Fig. 1). Respect to Normals, miR-223 showed a higher significant expression in all the other groups with the exception of Lymphoma, for which the difference was not significant, while miR-125b was up-regulated only in Glioblastoma, Medulloblastoma and Metastastasis, not showing any significant difference with Lymphoma and Benign.

RT-PCR results revealed that miR-223 was 3 fold (*p*-Value = 0, 03) more expressed in Glioblastomas compared to Medulloblastomas and that miR-125b was 3.9 fold higher in the Medulloblastoma respect to Glioblastoma group (*p*-Value = 0, 001). Therefore, we could consider miR-223 a differentiating miR for Glioblastoma and miR-125b a differentiating miR for Medulloblastoma in those CSF samples where there is a loss of miR-935 expression.

MiR-223 was up-regulated in Metastasis when compared to Normal and Lymphoma, while miR-125b was up-regulated in Metastasis when compared to Normal and Benign. When we compared Metastasis to Glioblastoma and Medulloblastoma, miR-223 expression was 3 folds higher in Glioblastoma, and was not deregulated in Medulloblastoma, while miR-125b expression was 4 fold increased in Medulloblastoma and was not deregulated in Glioblastoma. This, further confirms the specificity of miR-223 on identifying a Glioblastoma and for miR-125b on identifying a Medulloblastoma and, furthermore, may suggest that these two miRs could be part of the same pathway in lung and breast metastasis to the CNS.

## DISCUSSION

The aim of our study was to identify a CSF microRNA signature that could differentiate among CNS neoplasm. Our hope is to find new diagnostic biomarkers that can aid borderline or uncertain imaging results onto diagnosis of CNS malignancies, avoiding most invasive procedures such as stereotactic biopsy or biopsy. Therapeutic strategies could be planned in advance improving patients’ quality of life. Moreover, the identification of such biomarkers could help on finding alternative therapeutic targets.

Based on the knowledge that CSF is the CNS biological fluid, it flows only in the CNS, and it is easily collectable by a spinal tap at the lumbar cisternae level, we hypothesized that CSF would be the ideal biological fluid to find CNS biomarkers.

On the other hand, microRNAs have demonstrated to classify human cancers [16, 18] and to be very stable RNAs in CSF [20, 21]. CSF has also the advantage to contain fewer microRNAs than plasma or serum, which are, instead, flowing throughout the body and, thus, less tissue specific.

In our study, RNA of 34 cases of CNS neoplasms (Tab.1) and of 14 cases with no known malignancy or degenerative disease (a total of 82 samples) were processed by NanoString. Samples were allocated to one of the following groups according to their diagnosis: Normal, Benign, Lymphoma (CNS primary), Glioblastoma, Medulloblastoma, and Metastasis (from a primary Lung or Breast cancer). Data analysis allowed us to select the most commonly differentially expressed microRNAs in the majority of comparisons. RT-PCR validated five out of eight selected microRNAs: hsa-miR-451, -711, -935, -223, -125b.

Although NanoString identified differentially expressed microRNAs, it was not able to detect the true folds differences between groups. This could be due to the very small amount of CSF we processed: total RNA was extracted from 250 μL of CSF, enriched with carrier and spike-in RNAs. Thus, a measurement of 100 ng of total RNA would include the carrier, and other RNAs, decreasing the amount of microRNAs contained in each sample. Due to the limitation of CSF collection, we could not use higher CSF starting volumes. Therefore, NanoString is a very powerful technique for microRNA profiling, especially when dealing with small RNA quantities and rare samples.

This is the first study in which NanoString was successfully employed for CSF microRNA profiling.

Data were again confirmed by *in situ* hybridization experiments on normal and tumor CNS tissues (Fig. 2). *In situ* hybridization also showed that all the validated microRNAs were synthesized in CNS normal tissue and/or cancer cells, strengthening and confirming our hypothesis: CSF is the best biological fluid to find CNS biomarkers.

**Figure 3:**
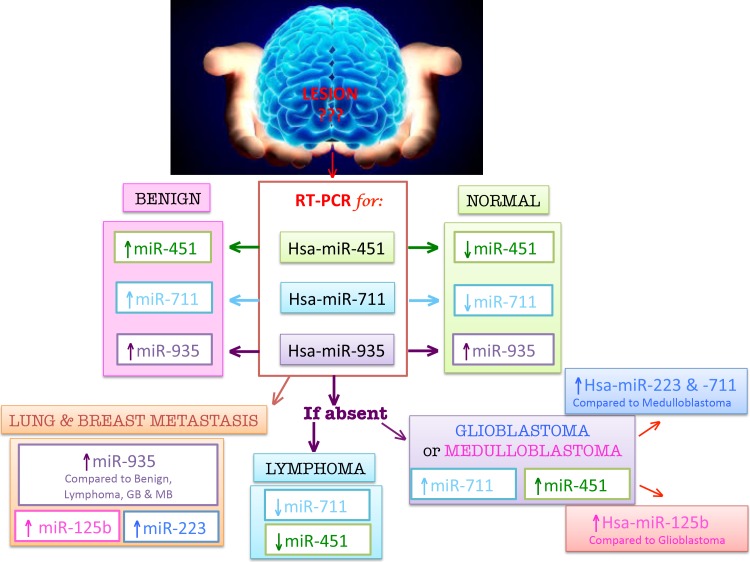
CSF Diagnostic Chart for CNS tumors In this figure, based on our results, we propose an hypothetical diagram to diagnose and differentiate neoplastic lesions with a simple RT-PCR on patients’ CSF RNA. As for any diagnostic RT-PCR procedure controls should be tested together with patients’ samples. Patients’ CSF RNA will be primarily tested for miR-451, -711, and -935 which will allow to discriminate among three groups: the Normal group (green box on the right) has the lowest expression of miR-451 respect to pathological groups, and a moderate expression of both miR-711 and -935; a moderate expression of all the three screened microRNAs (miR-451, miR-711 and miR-935) should address our diagnosis towards the group of Benign neoplasm (pink box toward the left), while, an low miR-451, an extremely low expression of miR-711 and the lack of miR-935, would suggest a primitive CNS Lymphoma (blue box). On the other hand, the total absence of miR-935 together with a moderate expression of miR-451 and of miR-711, would be strongly suggestive of Glioblastoma or Medulloblastoma. A differential diagnosis between Glioblastoma and Medulloblastoma will be achieved by testing the samples for miR-223 and 125b: compared to Medulloblastoma, in Glioblastoma we should find an increased expression of miR-223 and- 711 and a decreased expression of miR125b; compared to Glioblastoma, in Medulloblastoma we should find the reverse (high expression of miR-125b, lower expression of miR-223 and -711). The CSF RNA of a patient with a lung or breast cancer metastatic lesion to the brain will show the highest expression of miR-935 among tumoral lesions, and a variable high expression of miR-223 and -125b.

Additionally, based on RT-PCR comparisons, we found that hsa-miR-451, -711, -935, -223 and -125b were significantly differentially expressed between and among groups. Table 2 describes the fold changes and comparisons with significant p-Values, while Fig. 1 illustrates plotted differences among groups for each miR. Based on these data, we were able to draw an hypothetical diagnostic chart to follow in the event of a CNS lesion with an uncertain diagnosis (Fig. 3). The putative CSF sample RNA would be initially tested together with controls by RT-PCR for hsa-miR-451, hsa-miR-711 and hsa-miR-935. Our analysis identified hsa-miR-451 as the “normal miR” because its expression was down-regulated only in normal samples respect to CNS tumors, discriminating a normal from a neoplastic CSF. The difference was not significant when comparing Normal with Lymphomas, but as shown in Figure 1 and 2, miR-451 was evidently down-regulated in Normals respect to Lymphoma. This finding could be explained by numbers: 3 lymphoma cases/samples where compared to 14 Normal cases/samples.

Consistently with our findings, other authors reported that hsa-miR-451 was up-regulated in glioblastoma respect to normal tissues [22, 23], and that it induced proliferation and migration in glioma cells [24]. On the other hand, Cogswell et al. found that miR-451 is down-regulated in the CSF of Alzheimer's Disease patients compared to non-affected normal samples [25]. Thus, we can speculate that hsa-miR-451 shows the lowest CSF expression in Alzheimer's disease, a low expression in Normal, and the highest in benign and malignant CNS tumors. Considering that the differentiation between neoplastic lesions and degenerative diseases is an indication for a diagnostic stereotactic biopsy, with the exception of Lymphomas, CSF RT-PCR detection of high levels of hsa-miR-451 would be diagnostic for a CNS neoplasm.

CSF hsa-miR-711 showed the lowest expression in Lymphoma respect to malignant tumors. When we compared 9 benign cases to 3 lymphoma cases, hsa-miR-711 was 2.45 fold down-regulated in Lymphoma respect to Benign, with a borderline significance (*p*-Value = 0.058) that could be attributed to the low number of Lymphoma cases relative to Benign. *In situ* hybridization (Figure 2) clearly showed a decreased expression in Lymphoma tissue respect to the other neoplastic tissues, including Meningioma (benign CNS tumor). However, Ralfkiaer et al. found that hsa-miR-711 is up-regulated in cutaneous T-cell lymphoma respect to benign inflammatory skin lesions [26]. Furthermore, Freilich et al. observed a down-regulation of miR-711 during murine glial cells inflammatory switching from the resting to the phagocytic activation [27]. Tumor growth is always associated with an immune response that should limit cancer cells expansion and invasion. The CNS inflammatory response relays much more on the innate immunity (microglia, monocytes and macrophages) than the remaining parts of the body, thus it would be reasonable to expect differences in microRNAs’ expression patterns between a Primary CNS Lymphoma and Nodal Lymphoma. MiR-711 could aid the differential diagnosis between Lymphoma and Metastasis, Lymphoma and Glioblastoma, and Lymphoma and Medulloblastoma, while hsa-miR-935 would address us towards a definitive diagnosis. As shown in Table 2 and Figure 1 and 2, hsa-miR-935 was not expressed in Lymphoma respect to Normal, Benign, and Metastasis groups. Hence, hsa-miR-935 can differentiate a CNS Lymphoma from Normals and from a Benign and/or Metastatic lesion to the brain.

Recapitulating, our initial RT-PCR screening for hsa-miR-451, -711 and -935 was able to distinguish: 1) a Normal CSF by a low expression of miR-451 and a moderate expression of both miR-711 and -935; 2) a Benign neoplasm CSF by a moderate expression of all the three screened microRNAs; 3) a Lymphoma CSF by a low expression of miR-451, the lowest expression of miR-711 (relative to malignant tumors), and the lack of miR-935 expression.

A fourth combination of our signature expression pattern was characterized by a moderate expression of hsa-miR-451 and of hsa-miR-711 together with a total loss of hsa-miR-935. Hsa-miR-935 was not amplified by RT-PCR, nor its probe was staining Glioblastoma and Medulloblastoma samples (Fig. 2). Therefore, as illustrated in Figure 3, we can consider the absence of hsa-miR-935 as diagnostic not only for Lymphoma, but also for Glioblastoma or Medulloblastoma.

At this point of the chart, three microRNAs are differentiating between Glioblastoma and Medulloblastoma: hsa-miR-223, -125b and -711. Hsa-miR-223 and -711 were up-regulated in Glioblastoma relative to Medulloblastoma, while hsa-miR-125b was up-regulated in Medulloblastoma in comparison to Glioblastoma. MiR-125b is the most abundant microRNA in the brain. Its physiologic functions include neurogenesis and neural development through repression of several targets [28]. On the other hand, miR-125b seems to play a dual role in cancer: it promotes *in vitro* and *in vivo* proliferation and growth of glial and neuroblastoma cells [29–31], but behaves as a tumor suppressor in glioblastoma-associated endothelial cells, glioma stem cells and medulloblastoma [32–34]. Recently, Herinksen et al. identified a glioblastoma subgroup in which miR-125b up-regulation was associated with a prolonged patients’ survival [35]. Less is known on hsa-miR-223 functions in the CNS. Genovese et al. linked its expression to the proneural type glioblastoma [36] where it suppresses glial precursor proliferation through inactivation of NFIA *in vitro* and in human samples [37]. However, Huang et al. found that increased miR-223 expression promotes tumor growth and invasion in glioblastoma cell lines by targeting PAX6 [38].

In our study, hsa-miR-125b and hsa-miR-223 expression levels were evaluated in the comparison between Glioblastoma and Medulloblastoma, independently from the tumor suppressor or oncogenic role they might play. It is also true that, due to the limited number of samples, the observed differential expression could result from the comparison of specific prognostic subgroups, or from the comparison of non-homogeneous random classes of samples.

We tried to find microRNAs that could differentiate Metastasis from the rest of CNS neoplasm and/or between Lung and Breast Metastasis. As drawn in Figure 3, we could not find any specific metastatic microRNA, but we found a differential expression of our signature. We could differentiate lung and breast Metastasis to the CNS from: Normal by the up-regulation of hsa-miR-125b and -223, from Lymphoma by the up-regulation of hsa-miR-223 and -935, from Benign by the up-regulation of hsa-miR-125b and -935, from Medulloblastoma by the up-regulation of hsa-miR-935 and -125b, and from Glioblastoma by the up-regulation of hsa-miR-935 and -223.

Although our CSF microRNA signature needs to be tested on more samples and eventually amplified with additional pathology and prognostic classes to determine differentiating ranges of fold changes of expression among groups, our results are very promising.

We have been the first to employ a high-throughput microarray-like technique to identify a cancer CSF microRNA profile for CNS neoplasms. Data validation by *in situ* hybridization has proven that CSF signatures mirror CNS physiologic or pathologic condition. Our study defined a CSF diagnostic microRNA profile with good prospective of future clinical application.

## MATERIALS AND METHODS

### Samples

A total of 82 CSF samples were collected from the Regina Elena Institute, Rome, Italy and from The Ohio State University, Columbus, OH. Sixty eight samples came from patients with benign and malignant brain lesions (Table 1), while 14 from patients without brain disease. As shown in Table 1, in some cases, multiple samples were collected at different postsurgical time points from the same patients. Because of the semi-invasive nature of spinal fluid collection, we were not able to perform repetitive CSF withdrawn at the same time point and on all patients. We collected samples only when the access to the CSF was available for diagnostic or therapeutic reasons. All patients were informed and consented to the anonymous use of their CSF and clinical data, and tissues when available, for research purposes (IRE IRB N.CE44/14, OSU IRB N.2013H0178).

### RNA extraction

Two hundred and fifty μL of CSF were homogenized in 1–1, 5mL of Trizol reagent (Life Technologies Cat. 15596–018) and stored at −80°C. After thawing the samples on ice, 200 AttoMoles of spike-in RNA (Cel-miR-248) and 1 μL of RNA carrier (Ambion, Cat.4382878) were added. Trizol protocol was followed up to the recovering of the aqueous phase. The aqueous phase was then loaded on the RNA clean up and concentration kit columns (Norgen Cat. 23600). We followed the kit manufacturing instructions for total RNA extraction. To check RNA quality and yield test samples were analyzed by Agilent Eukaryote total RNA pico.

### NanoString nCounter assay

A total of 82 CSF samples were processed with NanoString. RNA concentration and quality were estimated by Nanodrop assay (Nanodrop Spectrophotometer 2000), and 100 ng were used as input for nCounter miRNA sample preparation reactions according to manufacturer's instructions (NanoStringTechnologies). Preparation of small RNA samples involves the ligation of a specific DNA tag onto the 3′ end of each mature miRNA. These tags are designed to normalize the melting temperatures of the miRNAs as well as to provide a unique identification for each miRNA species in the sample. The tagging is accomplished in a multiplexed ligation reaction using reverse-complementary bridge oligonucleotides to direct the ligation of each miRNA to its designated tag. Following the ligation reaction, excess tags and bridges are removed and the resulting material is hybridized with a panel of miRNA:tag-specific nCounter capture and barcoded reporter probes. Hybridization reactions were performed according to the manufacturer's instructions with 5 μL of the fivefold diluted sample preparation reaction. All hybridization reactions were incubated at 64°C for a minimum of 18 h. Hybridized probes were purified using the nCounter Prep Station (NanoString Technologies) following the manufacturer's instructions to remove excess capture and reporter probes and to immobilize transcript-specific ternary complexes on a streptavidincoated cartridge. Data collection was carried out on the nCounter Digital Analyzer (NanoString Technologies) following the manufacturer's instructions to count individual fluorescent barcodes and quantify target RNA molecules present in each sample. For each assay, a high-density scan (600 fields of view) was performed.

### NanoString data analysis

NanoString raw data was analyzed with nSolver^™^, a tool provided by NanoString Technologies. In particular, data was normalized by calculating the geometric mean of the top 100 miRNAs in all samples, as recommended by NanoString. P-values were calculated using the LIMMA package (Linear Models for Microarray Data) from the Bioconductor R project. The *p*-values were adjusted for multiple testing using the Benjamini and Hochberg method to control the False Discovery Rate (FDR). Raw data are available at NCBI GEO: GSE62381.

### Taqman stem-loop miRNA RT-PCR

Expression of mature single miRNAs was assessed in triplicate by the TaqMan Stem-loop miRNAassay (Applied Biosystems, Foster City, CA, USA), and normalized to Cel-miR-248 (Applied Biosystems) in all 82 samples. P-Values were calculated by one-tailed *t*-test. RT-PCR box plots are represent on Figure 1 as 2Λ^−ΔCt^ relative expression to Cel-miR-248. Means ± standard error of the mean (s.e.m.), *P < 0.05, by two-tailed Student's t test.

### *In situ* RNA hybridization

FFPE sections (Padua University) of primary central nervous system tumors (meningioma, glioblastoma multiforme, medulloblastoma, and lymphoma) and metastatic tumors to the brain (breast cancer, lung adenocarcinoma) were stained for miR-125b, miR-223, miR-451, miR-711, and miR-935. Five cases per pathologic sub-group were analyzed; further, 5 peri-lesional normal grey matter specimens were considered in the analysis. All probes were labeled with 5′-digoxigenin and synthesized by Exiqon (Denmark). *In situ* hybridization was performed as described, with minor modifications [39]. Negative controls included omission of the probe and the use of a scrambled LNA probe; U6 was used as positive control (Exiqon). Slides were counterstained in fast red solution.

## SUPPLEMENTARY TABLE


