# Supplementary material for: A differentially expressed set of microRNAs in cerebro-spinal fluid (CSF) can diagnose CNS malignancies
Source: Oncotarget. 2015 May 28;6(25):20829–39. doi: 10.18632/oncotarget.4096 (PMC4673232; doi:10.18632/oncotarget.4096)
Supplement: Supplementary file 1 [file oncotarget-06-20829-s001.pdf]

SUPPLEMENTAL FIGURE

Supplementary Table S1. Means and SDs of Figure 1

MEAN

Type	Normal	Benign	Lymph	Glio	Medullo	Met
miR-451	4.20	13.18	10.32	11.75	10.18	11.70
miR-711	8.78	7.70	6.40	9.54	8.86	8.17
miR-935	10.25	8.09	N.E.	N.E.	N.E.	8.94
miR-125b	3.89	6.31	7.82	9.72	11.69	9.48
miR-223	6.34	10.82	8.86	13.24	11.56	11.36

STANDARD DEVIATION

Type	Normal	Benign	Lymph	Glio	Medullo	Met
miR-451	2.55	5.86	6.32	5.74	4.80	2.86
miR-711	4.97	1.25	0.53	0.61	1.06	0.50
miR-935	4.74	1.26	N.E.	N.E.	N.E.	0.60
miR-125b	0.92	3.28	2.56	2.42	2.67	2.43
miR-223	5.48	2.90	0.97	1.89	1.90	2.71

N.E. Not Expressed
